# Male Breast Metastasis: A Case of Treatment-Emergent Neuroendocrine Prostate Cancer

**DOI:** 10.7759/cureus.24283

**Published:** 2022-04-19

**Authors:** Rita Sismeiro, Margarida Brito Monteiro, Catarina Negrão, Tiago Tomás, Marta Jonet

**Affiliations:** 1 Internal Medicine, Hospital Prof. Doutor Fernando Fonseca, Amadora, PRT; 2 Oncology, Hospital Prof. Doutor Fernando Fonseca, Amadora, PRT

**Keywords:** penile metastasis, lung metastasis, castration resistant metastatic prostate cancer, male breast cancers, treatment emergent neuroendocrine prostate cancer, breast metastasis, small cell carcinoma of the prostate

## Abstract

Treatment-emergent transformed neuroendocrine prostate cancer (NEPC) is a highly aggressive type of prostate cancer that may arise from typical adenocarcinoma of the prostate, which is associated with rapidly progressive disease involving visceral sites and refractoriness to hormonal therapy.

We present the case of a 74-year old male with a known history of stable prostate adenocarcinoma treated with transurethral prostate resection, local radiotherapy (RT), and androgen deprivation therapy (ADT) in 2020 who presented to the emergency room with complaints of shoulder and anterior chest pain, dyspnoea, and fatigue. Upon examination, a solid, adherent breast mass and infra-clavicular adenopathy were palpable. Thoracic computed tomography (CT) scan showed adenopathies in multiple thoracic chains, bilateral pulmonary nodular opacities, multiple osteolytic lesions, and bilateral enlargement of retro areolar tissue. A staging CT scan revealed further hepatic and penile lesions. Breast mass biopsy was compatible with small cell neuroendocrine cancer. Biopsies of the prostate, penis, lymph nodes, and bronchus were also performed. Histology of the prostate showed focal infiltration by the known adenocarcinoma while all others documented extensive infiltration by neuroendocrine carcinoma, whose morphology and immunohistochemical profile were identical to that of the breast.

This case highlights the challenges a diagnosis of neuroendocrine prostate cancer might pose, and the aggressiveness of this type of cancer, which frequently presents with advanced disease and is associated with poor outcomes.

## Introduction

Treatment-emergent transformed neuroendocrine prostate cancer (NEPC) is a highly aggressive type of prostate cancer that may arise from typical adenocarcinoma of the prostate, which is associated with rapidly progressive disease involving visceral sites and refractoriness to hormonal therapy [1-2]. NEPC tumors are also characterized by rapid disease progression, presence of lytic bone lesions, marked prostatic enlargement, and since they do not secrete prostate-specific antigen (PSA), disproportionally low levels of PSA in the setting of metastatic disease [3].

## Case presentation

We present the case of an obese 74-year-old male with a known history of prostate adenocarcinoma, with a Gleason score of 9 (5+4) and a tumor, node, metastasis (TNM) stage of T3aN1M0 at diagnosis in late 2019. He had been treated with transurethral prostate resection with an improvement in lower urinary tract obstructive symptoms and androgen deprivation therapy (ADT) in March 2020, and external hypofractionated intensity-modulated radiotherapy to the prostate, seminal vesicles, and pelvic lymph node chains in November 2020. He was a former smoker with a tobacco load of 10 pack-year units but had no other relevant past medical history.

The patient presented to the emergency room in April 2021 with one-week-long progressive complaints of shoulder and anterior pleuritic chest pain, effort dyspnoea, and fatigue. At admission, physical examination revealed prolongation of expiratory time and decreased vesicular sounds in both pulmonary bases and lower limb edema. Blood tests (Table 1) revealed normocytic normochromic anemia, slightly increased lactate dehydrogenase, slightly increased N-terminal pro-B-type natriuretic peptide (NT-proBNP), increased D-dimers, and increased PSA levels. All other laboratory results, including liver function tests, were normal. Bone alkaline phosphatase levels were not measured.

**Table 1:** Patient's laboratory findings at admission MCV - Mean Corpuscular Volume; MCH - Mean Corpuscular Hemoglobin; NT-proBNP - N-terminal pro b-type natriuretic peptide; PSA - Prostate-Specific antigen; LDH - Lactate Dehydrogenase; AST - Aspartate Aminotransferase; ALT - Alanine Aminotransferase; ALP - Alkaline Phosphatase; GGT - Gamma-Glutamyl Transferase

Laboratory parameters	Patient's results	Reference range
Hemoglobin	11.6 g/dL	13-17 g/dL
MCV	92 fL	80-100 fL
MCH	30.4 pg	27-33 pg
D-dimers	7676 µg/L	< 500 µg/L
NT-proBNP	524 pg/mL	< 486 pg/mL
PSA	13.7 ng/mL	< 6.5 ng/mL
LDH	287 U/L	135-225 U/L
AST	27 U/L	< 40 U/L
ALT	15 U/L	< 41 U/L
ALP	113.34 U/L	40-130 U/L
GGT	37 IU/L	< 60 IU/L
Total bilirubin	< 0.15 mg/dL	<= 1.20 mg/dL

**Figure 1:**
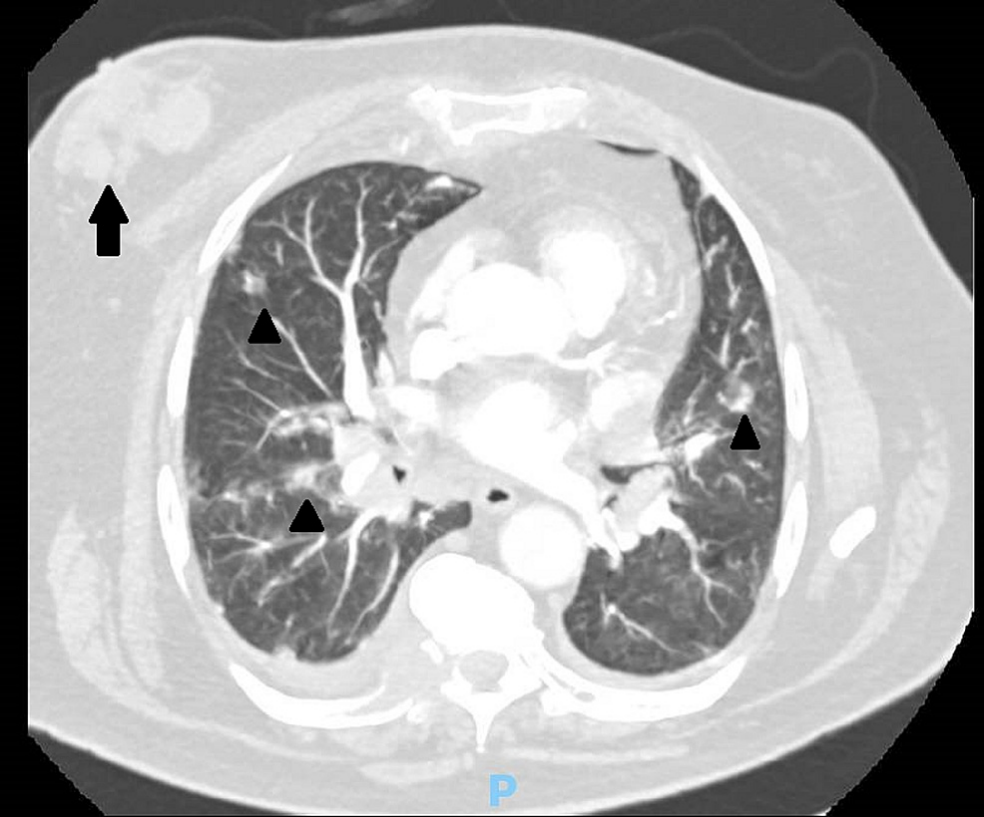
Chest CT angiography showing right retroareolar glandular tissue enlargement (arrow) and bilateral pulmonary nodules (arrowheads)

Thoracic computed tomography (CT) angiography scan ruled out pulmonary thromboembolism but showed several adenopathies in multiple thoracic chains, bilateral pulmonary nodular opacities, right pleural effusion, osteolytic lesions in multiple ribs and vertebrae, and bilateral enlargement of retroareolar glandular tissue that could be related to gynecomastia (Figure 1). Upon closer examination, left infra-clavicular adenopathy and a large, solid, hard, adherent mass of the right breast were palpable, which, according to the patient, had been increasing in size in the last four months, but it had been previously disregarded as gynecomastia.

**Figure 2:**
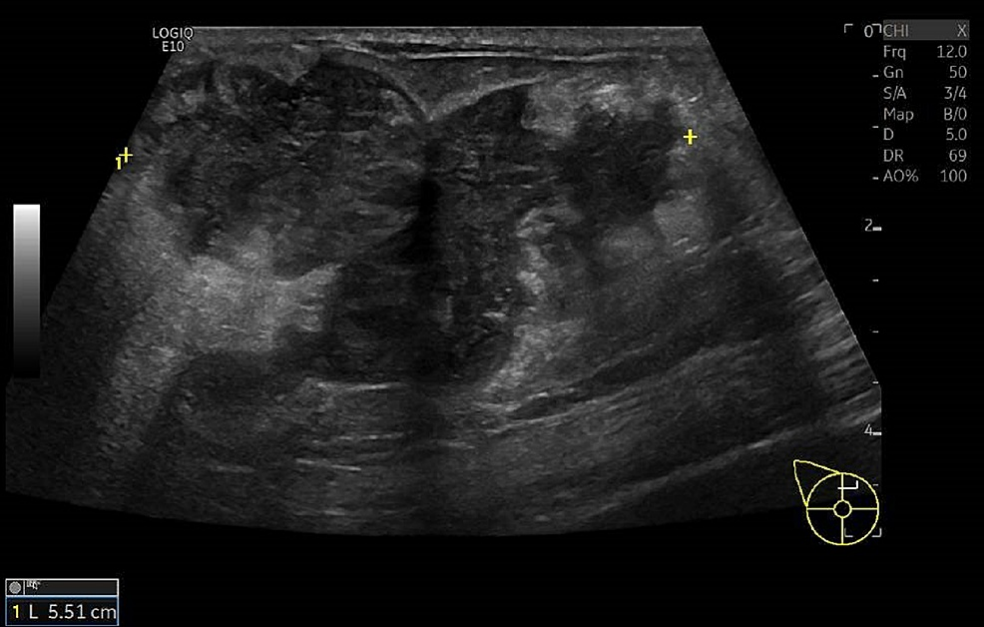
Echography of the right breast showing a large, hypoechoic, solid mass

Metastatic disease of unknown origin was suspected, and the patient was transferred to an Internal Medicine ward for further investigation. For etiological clarification, echography of the breast mass (Figure 2) and left infra-clavicular adenopathy were performed, which revealed a 55 x 32 mm, hypoechoic, solid mass with irregular outlines in the central and internal quadrants of the right breast compatible with an atypical lesion and a 30 x 21 mm left infra-clavicular, solid, hypoechoic nodule with irregular outlines that could be compatible with metastatic adenopathy or a primary tumor focus. Echography-guided biopsies of both lesions were performed.

Staging abdominal-pelvic CT scan revealed a right hepatic hypodense 20 mm nodule and a 30 mm penile root nodular lesion compatible with secondary deposits, left-sided hydronephrosis, largely increased prostatic volume, and bilateral para-aortic, iliac, and inguinal adenopathies. Cervical, lumbar, and sacral MRI and bone scintigraphy confirmed the presence of secondary osteolytic lesions in multiple vertebrae (C4, Th3, Th4, Th6-Th9, L2, and L3), several costal arches bilaterally, sternum and proximal epiphysis of both humeri. Disseminated metastatic cancer of the lung versus prostate was suspected, so echography-guided biopsies of the prostate and penile root were performed, as well as of the bronchus, via videobronchoscopy.

**Figure 3:**
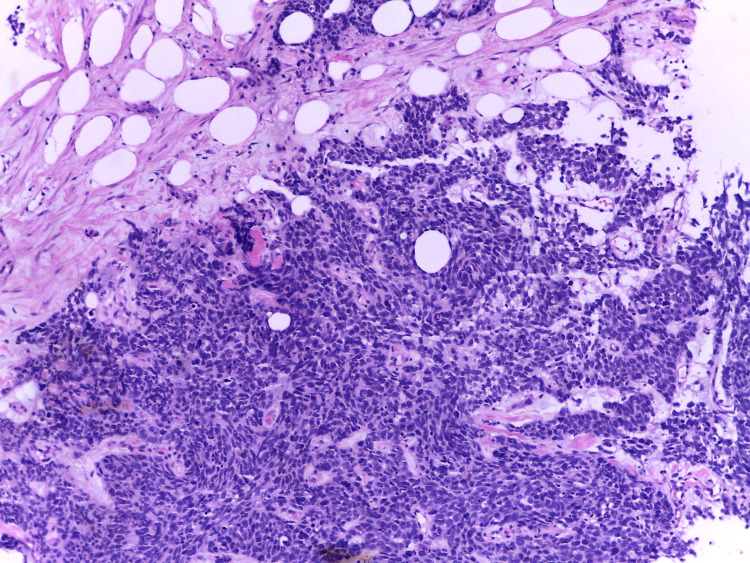
HE staining histology of the breast mass showing a neoplasm with diffuse growth, constituted by small to intermediate cells, with little cytoplasm, dull chromatin, and areas of necrosis HE - Hematoxylin & Eosin

Breast mass anatomopathology results (Figure 3) revealed a solid, malignant cellular pattern with areas of necrosis, with a neuroendocrine small cell morphologic pattern, negative for all markers usually expressed in breast cancer and positive for the following immunohistochemical markers: E-cadherin, epithelial membrane antigen (EMA), thyroid transcription factor 1 (TTF1), neural cell adhesion molecule (NCAM or CD56), synaptophysin (SYP), chromogranin A (CHGA), cytokeratin CAM5.2, and Ki-67.

**Figure 4:**
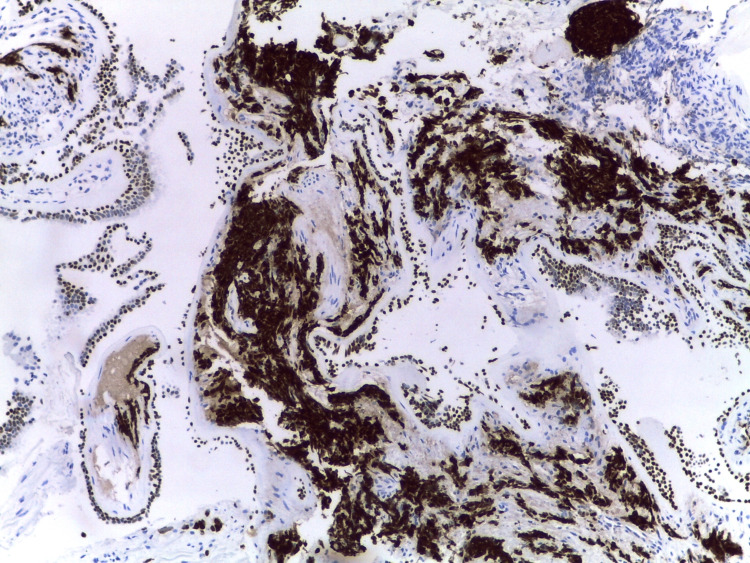
Immunohistochemical study of the bronchus showing TTF1 positivity TTF1 - Thyroid Transcription Factor 1

**Figure 5:**
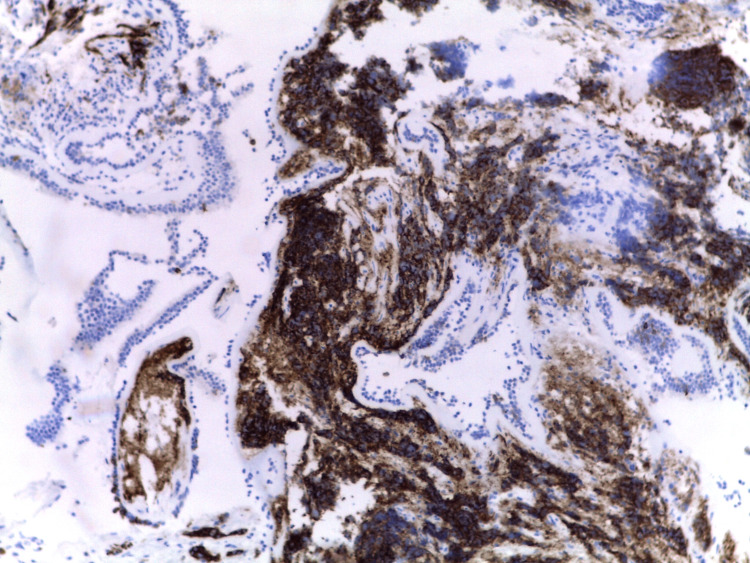
Immunohistochemical study of the bronchus showing CD56 positivity

**Figure 6:**
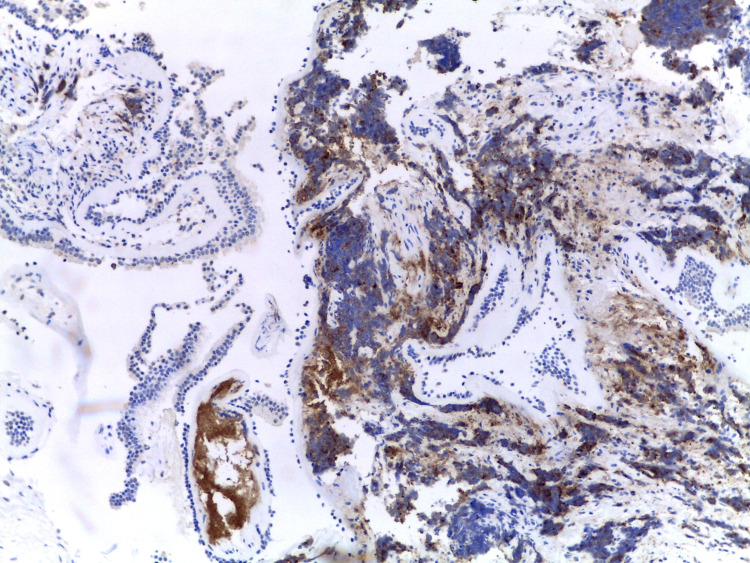
Immunohistochemical study of the bronchus showing synaptophysin positivity

Histology of the collected prostate tissue samples showed focal infiltration by the already documented adenocarcinoma, with marked morphologic changes related to the therapeutic effects of RT (karyomegaly, smudgy chromatin, and eosinophilic cytoplasm) but failed to document the presence of neuroendocrine cancer cells. However, all other biopsies’ results, namely that of the bronchus (Figures 4-6), documented extensive infiltration by small cell neuroendocrine carcinoma, whose morphology and immunohistochemical marker profile were identical to that of the breast.

Treatment-associated transformation of adenocarcinoma to NEPC was assumed as the primary diagnosis, and the patient was referred to an Oncology consultation, in order to start systemic treatment. He was also referred to antialgic radiotherapy (RT) for pain management of osteolytic bone lesions. Despite all diagnostic efforts, the patient had a very quick worsening of general status, which was aggravated by respiratory infection, and unfortunately passed away before targeted treatment was initiated.

## Discussion

NEPC is a rare variant of prostate cancer, with an overall estimated incidence of 0.35 cases per million per year, which, in most cases, presents with disseminated disease at the time of diagnosis [4]. This type of cancer can arise de novo, but it can also be a late manifestation connected to the treatment of prostate adenocarcinoma [3]. Due to the treatment-associated inhibition of androgen signaling pathways, prostate adenocarcinoma cells may undergo a lineage switch and start exhibiting characteristics of neuroendocrine cells, such as expression of neuronal markers including CHGA and SYP, in a process known as neuroendocrine differentiation [5].

Treatment-emergent NEPC tumors are characterized by the presence of lytic bone lesions, marked prostatic enlargement, and since they are not able to secrete it, disproportionally low levels of PSA in the setting of metastatic disease [3]. Our patient’s prostate was markedly enlarged, as per the pelvic CT results, but his PSA level of 13.7 ng/mL was higher than the mean PSA reported in this variant of prostate cancer, though still within a range found in 14% of cases [4]. Multiple widespread osteolytic lesions were also found, as stated in both vertebral column MRI and scintigraphy results.

Visceral metastases are common in NEPC, mostly to the liver, lung, and central nervous system [2,5]. These sites, however, are also commonly involved in metastatic neuroendocrine cancers of pulmonary origin [6], so we were faced with our first differential diagnosis challenge. Metastasis to the breast of extra-mammary tumors is an extremely rare occurrence but has been reported in neuroendocrine cancers of both origins [7-8]. Therefore, this finding, despite being incredibly rare, didn’t help us in pointing toward one or the other direction either.

In our patient’s case, among all tested immunohistochemical markers, positivity for TTF-1 was observed. This posed a further differential diagnostic challenge since TTF-1 is considered to be a specific marker for primary lung small cell carcinoma [9-10], but it can also be found in small cell neuroendocrine tumors of other origins, namely, the prostate, therefore limiting its use in distinguishing these two entities [10]. Considering the patient’s history of ADT-treated prostate adenocarcinoma and since the EMA and CAM5.2 markers are found in normal prostate samples, and common neuroendocrine markers, such as CHGA, SYP, and CD65, were present [10], the diagnostic hypothesis of treatment-associated small cell neuroendocrine cancer of prostatic origin was favored.

This case is a great example of the rapid disease progression and poor prognosis associated with this diagnosis [1-3]; only six months separate the patient first noticing the lump in his breast till the time of his passing. By the time of diagnosis, this patient had widespread distant organ involvement to the breast, lungs, liver, bone, penis, and lymph nodes. Despite being approached with the intent to treat, his general health status rapidly worsened, aggravated by a respiratory infection, which culminated in his death.

## Conclusions

Neuroendocrine prostate cancer can arise de novo or as the treatment-associated transformation of an established adenocarcinoma. Treatment-emergent neuroendocrine prostate cancer is associated with rapidly progressive disease and poor patient outcomes.

Differentiating the origin of metastatic neuroendocrine tumors can prove to be a tremendous challenge since the available immunohistochemical markers commonly used in the diagnosis of these cancer variants are still too unspecific.
